# Exciton *g*-factors in monolayer and bilayer WSe_2_ from experiment and theory

**DOI:** 10.1038/s41467-020-18019-1

**Published:** 2020-09-10

**Authors:** Jonathan Förste, Nikita V. Tepliakov, Stanislav Yu. Kruchinin, Jessica Lindlau, Victor Funk, Michael Förg, Kenji Watanabe, Takashi Taniguchi, Anvar S. Baimuratov, Alexander Högele

**Affiliations:** 1 Fakultät für Physik, Munich Quantum Center, and Center for NanoScience (CeNS), Ludwig-Maximilians-Universität München, 80539 München, Germany; 2 Information Optical Technologies Center, ITMO University, Saint Petersburg, 197101 Russia; 3 Center for Computational Materials Sciences, Faculty of Physics, University of Vienna, 1090 Vienna, Austria; 4 Research Center for Functional Materials, National Institute for Materials Science, Tsukuba, 305-0044 Japan; 5 International Center for Materials Nanoarchitectonics, National Institute for Materials Science, Tsukuba, 305-0044 Japan; 6 Munich Center for Quantum Science and Technology (MCQST), 80799 München, Germany

**Keywords:** Magneto-optics, Condensed-matter physics, Two-dimensional materials

## Abstract

The optical properties of monolayer and bilayer transition metal dichalcogenide semiconductors are governed by excitons in different spin and valley configurations, providing versatile aspects for van der Waals heterostructures and devices. Here, we present experimental and theoretical studies of exciton energy splittings in external magnetic field in neutral and charged WSe_2_ monolayer and bilayer crystals embedded in a field effect device for active doping control. We develop theoretical methods to calculate the exciton *g*-factors from first principles for all possible spin-valley configurations of excitons in monolayer and bilayer WSe_2_ including valley-indirect excitons. Our theoretical and experimental findings shed light on some of the characteristic photoluminescence peaks observed for monolayer and bilayer WSe_2_. In more general terms, the theoretical aspects of our work provide additional means for the characterization of single and few-layer transition metal dichalcogenides, as well as their heterostructures, in the presence of external magnetic fields.

## Abstract (web summary)

Excitons in various spin and valley configurations control the optical properties of ultrathin transition metal dichalcogenides. Here, the authors develop theoretical and experimental methods to determine the exciton *g*-factors for all possible spin-valley configurations of excitons in monolayer and bilayer WSe_2_, including valley-indirect excitons.

## Introduction

Monolayer (ML) and bilayer (BL) transition metal dichalcogenides (TMDs) such as WSe_2_ represent semiconductor building blocks for novel van der Waals heterostructures. By virtue of sizable light–matter coupling governed by excitons^1^, they exhibit versatile potential for applications in photonics and optoelectronics^2,3^, opto-valleytronics^4,5^, and polaritonics^6^. Most recently, the optical interface to TMDs has been instrumental for the observation of strongly correlated electron phenomena in twisted homobilayer and heterobilayer moiré systems^7–9^.

The key to further developments of van der Waals heterostructures for fundamental studies and practical devices using TMD MLs and BLs is the detailed understanding of their optical properties. While substantial understanding of zero-momentum excitons in ML and BL WSe_2_ has been established^1^, some important aspects remain subject of debate^10^. This holds, in particular, for valley-dark excitons with finite center-of-mass momentum that escape direct optical probes by virtue of momentum mismatch with photons. In MLs, they complement the notion of intravalley spin-bright and spin-dark excitons^1^, and they entirely dominate the photoluminescence (PL) from the lowest-energy states in native homobilayers of WSe_2_ (ref. ^11^).

Within the realm of optical spectroscopy techniques, magneto-spectroscopy provides means for studying the exciton spin and valley degrees of freedom. Magneto-luminescence experiments on ML WSe_2_ in the presence of out-of-plane and in-plane magnetic fields, for instance, have been used to quantify the valley Zeeman splitting of bright excitons^12–17^ or to brighten spin-dark excitons^18–20^, respectively. To date, however, a rigorous assignment of exciton *g*-factors to intervalley excitons with finite momentum falls short mainly due to the lack of theoretical predictions^10^.

In this work, we develop theoretical methods to evaluate *g*-factors for excitons in different spin and valley configurations, and provide explicit values for WSe_2_ ML and BL excitons composed from electron and hole states away from high symmetry points of the first Brillouin zone. Our calculations go beyond the existing tight-binding models by employing the density functional theory (DFT). We compare our theoretical results with experimentally determined *g*-factors of intravalley excitons and use them to interpret ambiguous peaks in the PL spectra of ML and BL WSe_2_ attributed to intervalley excitons. The technique can be expanded to other materials like WS_2_ with similarly complex spectra^21^ and large *g*-factors of ML^17^ and BL^22^ excitons.

## Results

### Magneto-luminescence spectroscopy of charge-controlled ML and BL WSe_2_

**Fig. 1:**
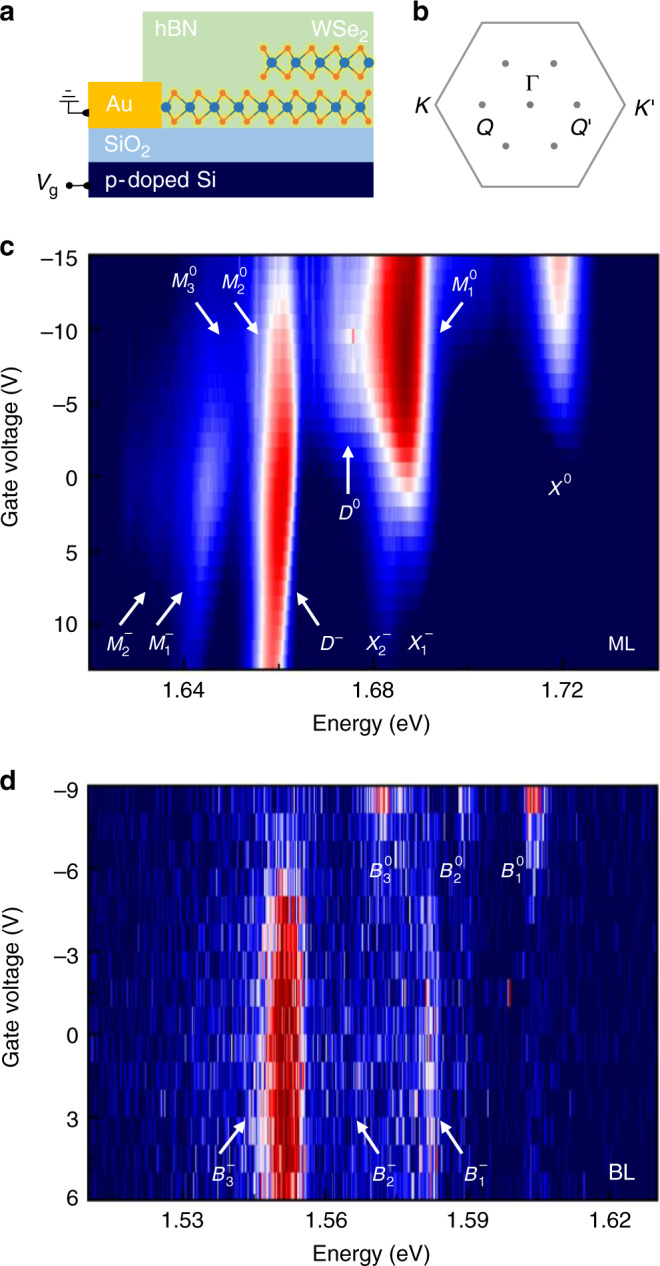
Sample layout and charge-doping control of monolayer and bilayer WSe_2_. **a** Schematics of the field-effect device with monolayer and bilayer WSe_2_ encapsulated in hBN. **b** First Brillouin zone with high symmetry points. **c**, **d** Logarithmic false-color plots of the photoluminescence as a function of the gate voltage, recorded at representative positions of monolayer and bilayer WSe_2_, respectively. The monolayer features characteristic photoluminescence of neutral bright (*X*^0^) and dark (*D*^0^) excitons, as well as the negatively charged bright trion doublet (${X}_{1}^{-}$ and ${X}_{2}^{-}$) and dark trion (*D*^−^). All other peaks of monolayer (*M*) and bilayer (*B*) photoluminescence are labeled according to their charge state in the superscript and an increasing subscript number.

In our experiments, we used a field-effect heterostructure based on an exfoliated WSe_2_ crystal with extended ML and BL regions encapsulated in hexagonal boron nitride (hBN). The device layout is shown schematically in Fig. 1a (see the Methods section for details) and the first Brillouin zone of ML and BL WSe_2_ with most relevant points in Fig. 1b. The sample was cooled down to 3.2 K, and the PL was probed as a function of voltage-controlled doping with laser excitation at 1.85 eV and powers below the regimes of neutral and charged biexcitons^23–26^. Magneto-luminescence experiments were performed in Faraday configuration with a bi-directional solenoid at magnetic fields of up to 9 T (see the Methods section for experimental details).

The evolution of the PL with the gate voltage is shown in Fig. 1c, d for representative spots of ML and BL regions, respectively. In Fig. 1c, the ML reaches the intrinsic limit at gate voltages <−5 V consistent with residual n-doping of the exfoliated crystal^27,28^. The neutral regime is characterized by the bright exciton PL (*X*^0^) at 1.72 eV and a series of red-shifted peaks that we label as ${M}_{1}^{0}$, ${M}_{2}^{0}$, and ${M}_{3}^{0}$. None of these peaks with respective red-shifts of 35, 60 and 75 meV from the bright exciton peak is to be attributed to the PL of dark excitons (*D*^0^) with 42 meV red-shift^20,29,30^. In our sample, this feature is a rather weak shoulder at the low-energy side of ${M}_{1}^{0}$. At positive gate voltages, the ML is charged with electrons and thus exhibits the characteristic signatures of a bright trion doublet (${X}_{1}^{-}$ and ${X}_{2}^{-}$) split by the exchange energy of  ~6 meV (ref. ^28^), the dark trion (*D*^−^) at 28 meV red-shift from ${X}_{1}^{-}$ (refs. ^31–34^), and a series of low-energy peaks dominated by the peak ${M}_{1}^{-}$ at 44 meV red-shift^33,34^.

The PL from the BL region in Fig. 1d is characterized by a multi-peak structure, >100 meV below *X*^0^. It exhibits the same limits of charge neutrality and electron doping as a function of the gate voltage, consistent with the charging behavior of the ML in Fig. 1c. The BL peaks, labeled by an increasing subscript number with decreasing peak energy as ${B}_{1}^{0}$ through ${B}_{3}^{0}$ and ${B}_{1}^{-}$ through ${B}_{3}^{-}$ in the neutral and negative regime, respectively, correspond to phonon sidebands of neutral and charged momentum-indirect excitons with a global red-shift of 22 meV at about  −7 V (ref. ^11^) in Fig. 1d. According to the single-particle band structure of BL WSe_2_ (refs. ^35,36^), the field-induced electron concentration is accommodated at the conduction band edge by the six inequivalent *Q*-valleys. However, the nature of the hole states that constitute the lowest-energy momentum-dark excitons as long-lived reservoirs of phonon-assisted PL remains ambiguous. The energetic proximity of the valance band edge states at *K* and Γ in BL WSe_2_ (ref. ^37^) renders *QK* and *Q*Γ excitons and trions (composed from electrons at *Q* and holes at *K* or Γ) nearly degenerate, which in turn complicates their energetic ordering^11^.

**Fig. 2:**
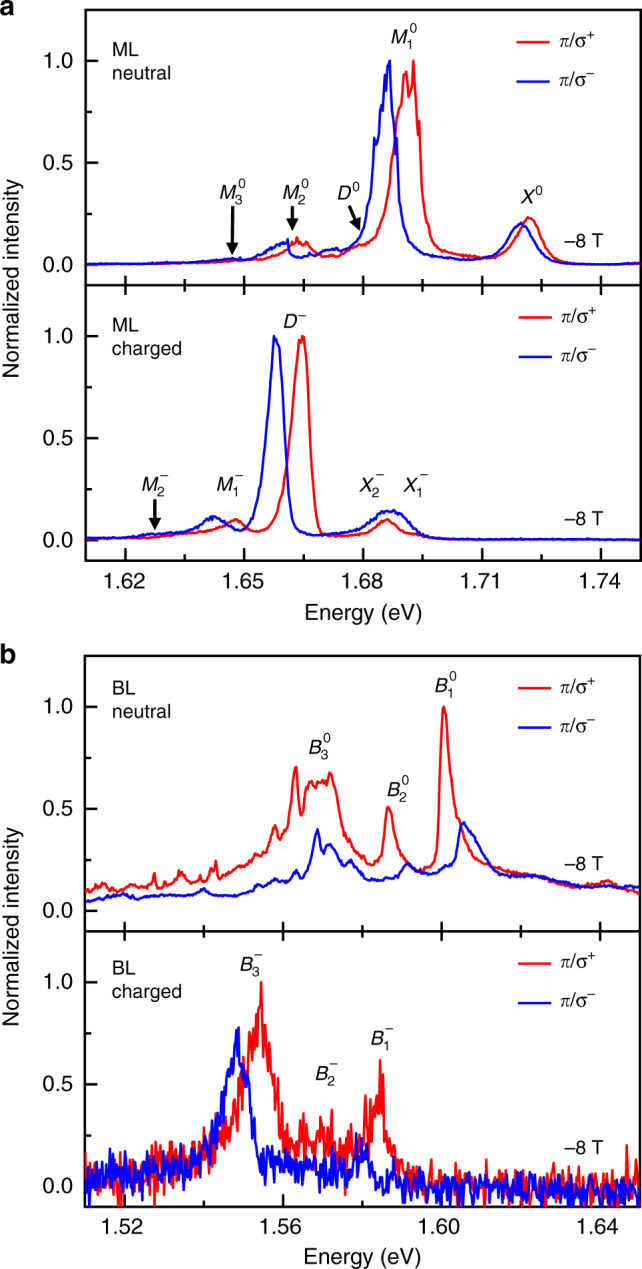
Magneto-luminescence spectroscopy of charge-controlled monolayer and bilayer WSe_2_. **a**, **b** Photoluminescence spectra of monolayer and bilayer WSe_2_, respectively, in a perpendicular magnetic field of  −8 T. The neutral and negatively charged regimes are shown in the top and bottom panels, respectively. The spectra were recorded with linearly polarized excitation (*π*) and circularly polarized detection (*σ*^+^ and *σ*^−^).

To examine the origin of the BL peaks and to shed light on the nature of ML peaks with ambiguous or partly controversial interpretation, we performed magneto-spectroscopy in the two well-defined limits of charge neutrality and negative doping. The external magnetic field *B* was applied along the *z*-axis perpendicular to the sample. It removes the valley degeneracy and splits the exciton reservoirs by their characteristic Zeeman energies proportional to the exciton *g*-factor in WSe_2_ (refs. ^12–17^). The respective polarization-contrasting spectra recorded at  −8 T under linearly polarized excitation (*π*) and circularly polarized detection (*σ*^+^ and *σ*^−^) for the neutral (negatively charged) ML and BL are shown in the top (bottom) panel of Fig. 2a, b.

**Fig. 3:**
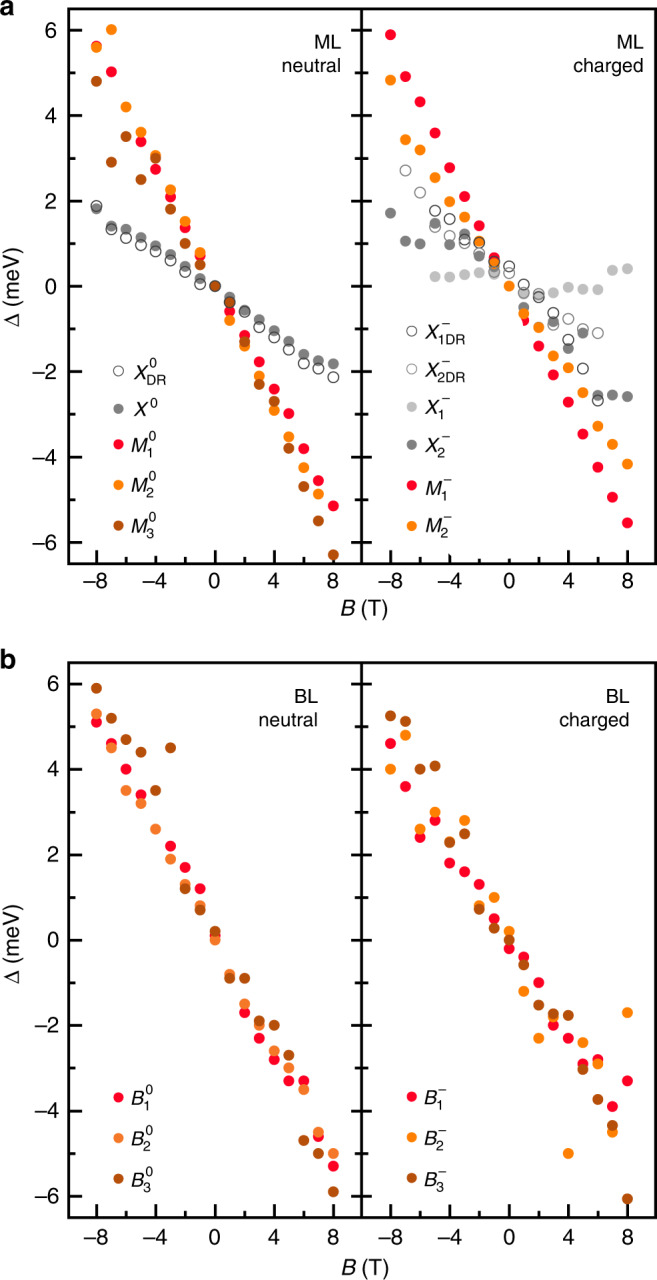
Valley Zeeman splittings in charge-controlled monolayer and bilayer WSe_2_. **a**, **b** Valley Zeeman splitting Δ as a function of the magnetic field for the photoluminescence peaks (closed circles) of monolayer and bilayer WSe_2_ in the neutral (left panel) and negatively charged (right panel) regimes. Complementary data (open circles) were obtained from polarization-resolved reflectivity.

At each magnetic field, we quantified the experimental Zeeman splitting for every PL peak as the energy difference Δ = *E*^+^ − *E*^−^ between the peak energies *E*^+^ and *E*^−^ recorded under *σ*^+^ and *σ*^−^ polarized detection. The left and right panels of Fig. 3a, b show Δ as a function of the magnetic field for all peaks of the neutral and negatively charged ML and BL, respectively. The set of data derived from magneto-PL measurements was complemented for *X*^0^, ${X}_{1}^{-}$, and ${X}_{2}^{-}$ by performing magneto-reflectivity under circular excitation and detection. The corresponding experimental exciton *g*-factors, obtained from Δ = *gμ*_B_*B* as the slopes of best linear fits to the data in Fig. 3 scaled by the Bohr magneton *μ*_B_, are summarized in Table 1. The negative sign of the *g*-factors reflects the energy ordering of exciton states that exhibit higher (lower) energy for *σ*^−^ (*σ*^+^) polarized PL peaks at positive magnetic fields.

**Table 1:** Experimental exciton *g*-factors in charge-controlled monolayer and bilayer WSe_2_.

ML				BL		
*X*^0^	${M}_{1}^{0}$	${M}_{2}^{0}$	${M}_{3}^{0}$	${B}_{1}^{0}$	${B}_{2}^{0}$	${B}_{3}^{0}$
−4.1	−11.5	−12.6	−11.4	−11.4	−10.8	−12.8
±0.1	±0.1	±0.2	±0.4	±0.2	±0.1	±0.2
${X}_{1}^{-}$	${X}_{2}^{-}$	*D*^−^	${M}_{1}^{-}$	${B}_{1}^{-}$	${B}_{2}^{-}$	${B}_{3}^{-}$
−4.6	−1.3	−12.2	−9.0	−9.1	−9.8	−11.5
±0.3	±0.3	±0.1	±0.1	±0.3	±1.0	±0.4

Experimental exciton *g*-factors obtained from magneto-luminescence (^a^complementary data from magneto-reflectivity) of neutral and negatively charged monolayer and bilayer WSe_2_.

^a^*X*^0^: −4.3 ± 0.1; $${X}_{1}^{-}:-4.7\pm 0.3$$; $${X}_{2}^{-}:-6.5\pm 0.4$$.

In ML WSe_2_, the *g*-factors of both neutral and negatively charged excitons with the corresponding PL peaks *X*^0^, *D*^0^, ${X}_{1}^{-}$, ${X}_{2}^{-}$, and *D*^−^ have been established in previous experiments on a wide range of different samples^12–20,31–34^. Our results for the bright exciton and the trion doublet in Table 1 agree well with these reports if we discard the magneto-luminescence result for ${X}_{1}^{-}$ that is compromised by both a vanishingly small PL intensity at high magnetic fields and the relatively broad linewidth of 6 meV in our sample. Due to this inhomogeneous broadening, we are unable to track the dispersion of the relatively weak spin-dark exciton peak *D*^0^, with *g*-factors ranging between 9.1 and 9.9 in previous reports^20,31,33,34^ nor its chiral-phonon replicum with the same *g*-factor at 65 meV red-shift from *X*^0^ (refs. ^33,34,38^). The signature of the latter is overwhelmed in our spectra by the peak ${M}_{2}^{0}$ with 60 meV red-shift and a *g*-factor of  −12.9 ± 0.7 in agreement with values reported from samples with spectrally narrow PL^33,34^. The red-most peak ${M}_{3}^{0}$ features the same *g*-factor within the experimental error bars as ${M}_{1}^{0}$, suggesting a joint reservoir as their origin. The negatively charged trion *D*^−^ was reported to have the same *g*-factor as its neutral counterpart^31–34^, whereas we determine  −12.2 ± 0.1. The agreement with previous reports is better for the peak ${M}_{1}^{-}$ with a *g*-factor of  −9.0 ± 0.1 that is supposed to be a phonon sideband of *D*^−^ (refs. ^33,34^). The latter studies also reported an intense PL peak between ${M}_{1}^{-}$ and *D*^−^ with a remarkably small *g*-factor of  −4.1 (ref. ^33^) and  −3.4 (ref. ^34^). This peak of unidentified origin is missing in our spectra from the negative doping regime.

There are other peaks in ML WSe_2_ without conclusive assignment, and in particular ${M}_{1}^{0}$ has received controversial interpretation as phonon-assisted PL from virtual trions^39^, phonon sidebands of momentum-dark *Q*-excitons^21^, or zero-phonon PL of finite-momentum excitons in spin-like configuration^34^ that we denote as ${K}_{L}^{\prime}$. Due to the lack of theory for the *g*-factors of excitons with finite center-of-mass momentum, the task of confronting the competing hypotheses with the characteristic valley Zeeman splittings of controversial ML peaks has remained elusive. The same shortcoming holds for both neutral and charged BL excitons with finite center-of-mass momentum. To shed additional light on the nature of PL peaks in both ML and BL WSe_2_, we calculate in the following the *g*-factors for excitons in different spin and valley configurations from DFT.

### Ab initio calculations of exciton *g*-factors

We consider a crystal electron in a Bloch state ${\psi }_{n{\bf{k}}}({\bf{r}})={S}^{-1/2}\exp ({\rm{i}}{\bf{kr}}){u}_{n{\bf{k}}}({\bf{r}})$ with energy *E*_*n***k**_, where *n* is the band number, **k** is the wave vector, *u*_*n***k**_(**r**) is the periodic Bloch amplitude, and *S* is the normalization area. In the presence of a weak perturbation by a static magnetic field **B**, the first-order correction to the electron energy is proportional to **B** and given by^40^:

1 $${V}_{n}({\bf{k}})={\mu }_{{\rm{B}}}{\bf{B}}[{g}_{0}{\bf{s}}+{{\bf{L}}}_{n}({\bf{k}})],$$

where *μ*_B_ = ∣*e*∣*ℏ*/(2*m*_0_*c*) is the Bohr magneton, *e* and *m*_0_ are the charge and mass of the free electron, *ℏ* is the Planck constant, and *c* is the speed of light. The expression in square brackets is usually called the effective magnetic moment^41,42^, which contains both spin and orbital contributions. In particular, the first term is proportional to the free electron Landé factor *g*_0_ ≃ 2 and the spin angular momentum **s** =  ***σ***/2, where ***σ*** denotes the Pauli matrix.

The second term, ${{\bf{L}}}_{n}({\bf{k}})=\left\langle {\psi }_{n{\bf{k}}}({\bf{r}})\right|{\bf{L}}\left|{\psi }_{n{\bf{k}}}({\bf{r}})\right\rangle$, is the orbital angular momentum with the operator **L** = *ℏ*^−1^[**r** × **p**]. To obtain its matrix elements, one can reduce the calculation to the interband matrix elements of the space coordinate operator **r**^14,41–43^:

2 $${{\bf{L}}}_{n}({\bf{k}})=\frac{{m}_{0}}{{\rm{i}}{\hslash }^{2}}{\sum \limits_{m\ne n}}[{{\boldsymbol{\xi }}}_{nm}({\bf{k}})\times {{\boldsymbol{\xi }}}_{mn}({\bf{k}})]({E}_{n{\bf{k}}}-{E}_{m{\bf{k}}}),$$

where *m* is the sum over all bands with energy *E*_*n***k**_ but the band of interest, and ${{\boldsymbol{\xi }}}_{nm}({\bf{k}})={\rm{i}}\left\langle {u}_{n{\bf{k}}}({\bf{r}})\right|\partial /\partial {\bf{k}}\left|{u}_{m{\bf{k}}}({\bf{r}})\right\rangle$ is the interband matrix element of the crystal coordinate operator.

In the following, we restrict our analysis to the orientation of the magnetic field along the *z*-axis and define the electron Zeeman splitting as the difference between the energy of the electron state with wave vector  +**k** and spin projection  +*s* along the *z*-axis and the state with  −**k** and  −*s* as:

3 $${\Delta }_{n}({\bf{k}})={V}_{n}(+{\bf{k}})-{V}_{n}(-{\bf{k}})=2{\mu }_{{\rm{B}}}B[{g}_{0}s+{L}_{n}({\bf{k}})].$$

Thus the electron *g*-factor of Bloch electrons in the *n*th band can be written as:

4 $${g}_{n}({\bf{k}})=\frac{{\Delta }_{n}({\bf{k}})}{{\mu }_{{\rm{B}}}B}=\pm {g}_{0}+2{L}_{n}({\bf{k}})$$

with  +(−) for *s* = +1/2 (−1/2) corresponding to spin up (down) projections along *z* denoted as ↑ (↓), and the explicit expression for the *z*-component of the orbital angular momentum:

5 $${L}_{n}({\bf{k}})=\frac{{m}_{0}}{{\hslash }^{2}}{\sum\limits_{m\ne n}}\left[{\left|{\xi }_{mn}^{(-)}({\bf{k}})\right|}^{2}-{\left|{\xi }_{mn}^{(+)}({\bf{k}})\right|}^{2}\right]({E}_{n{\bf{k}}}-{E}_{m{\bf{k}}}),$$

where $${\xi }_{mn}^{(\pm )}=({\xi }_{mn}^{(x)}\pm {\rm{i}}{\xi }_{mn}^{(y)})/\sqrt{2}$$.

To calculate the contributions of the conduction (*c*) band electron with **k**_*c*_, *s*_*c*_ and the hole (*h*) with **k**_*h*_, *s*_*h*_ to the exciton *g*-factor, we neglect electron–hole Coulomb interactions^14,44^. In this case, the exciton Zeeman splitting simplifies to the sum of the Zeeman splittings of the electron and the hole. Using time reversal symmetry that relates the spin and wave vector of the hole to the corresponding spin and wave vector of the empty electron state in the valence (*v*) band (*s*_*h*_ = −*s*_*v*_ and **k**_*h*_ =−**k**_*v*_), we obtain the exciton *g*-factor as

6 $${g}^{(cv)}({{\bf{k}}}_{c},{{\bf{k}}}_{v})={g}_{c}({{\bf{k}}}_{c})-{g}_{v}({{\bf{k}}}_{v}).$$

Finally, by reference to the valence band electron with **k**_*v*_ = *K* or Γ with spin-up projection *s*_*v*_ = +1/2, we discriminate spin-like (*L*) excitons (with *s*_*c*_ = *s*_*v*_) from spin-unlike (*U*) excitons (with *s*_*c*_ = −*s*_*v*_). Their respective exciton *g*-factors are given by:

7 $${g}_{L}^{(cv)}({{\bf{k}}}_{c},{{\bf{k}}}_{v})=2[{L}_{c}({{\bf{k}}}_{c})-{L}_{v}({{\bf{k}}}_{v})],$$

8 $${g}_{U}^{(cv)}({{\bf{k}}}_{c},{{\bf{k}}}_{v})=2[{L}_{c}({{\bf{k}}}_{c})-{L}_{v}({{\bf{k}}}_{v})]-2{g}_{0}.$$

**Fig. 4:**
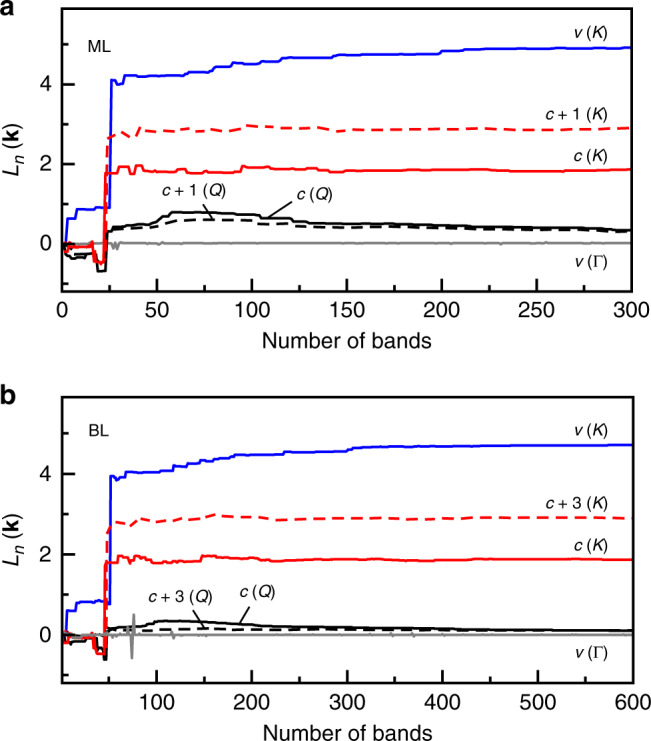
Electron orbital angular momentum in monolayer and bilayer WSe_2_ from DFT. **a**, **b** Electron orbital angular momentum from DFT calculations for the highest valence bands at *K* and Γ and the lowest conduction bands at *K* and *Q* in monolayer and bilayer WSe_2_, respectively.

Using Eqs. (7) and (8), we calculate in the following the exciton *g*-factors from the orbital angular momenta *L*_*c*_(**k**_*c*_) and *L*_*v*_(**k**_*v*_) of conduction and valence bands obtained from Eq. (5) within DFT calculations on the Γ-centered $\overrightarrow{k}$ grid of 12 × 12 divisions with 300 (600) bands (see the Methods section for details of DFT calculations). In Fig. 4a, b, we show the convergence of the orbital angular momenta *L*_*n*_(**k**) within our ML and BL calculations as a function of the number of bands taken into account in the sum of Eq. (5). For the ML, Fig. 4a shows the results for the top-most valence band state *v* at *K* (blue solid line) and the highest valence band state *v* at Γ (gray solid line), as well as the two lowest conduction band states *c* and *c* + 1 at *K* and *Q* (red and black solid and dashed lines). As the BL bands are doubly degenerate, each **k**-point of the Brillouin zone has at least two bands with *L*_*n*_(**k**) =  *L*_*n*+1_(**k**) or *L*_*n*_(**k**) = *L*_*n*−1_(**k**). For the BL in Fig. 4b, we consider the same **k**-points as for the ML and show the corresponding bands where the orbital angular momenta have the same sign as in the ML case of Fig. 4a.

For the orbital angular momenta of these states, convergence is observed above 275 and 550 bands in the case of ML and BL in Fig. 4a, b, respectively, with the factor of two difference related to the doubled number of atoms in BL calculations. We note that the values for the valence band states at Γ must vanish by symmetry arguments, whereas our numerical calculations yield  ±0.01 for both ML and BL. This marginal discrepancy is due to a finite number of bands taken into account and can be used to estimate the precision of our numerical calculations. The corresponding bound on the absolute error of the exciton *g*-factors from DFT, given explicitly in Table 2 for selected exciton configurations, is thus in the order of  ±0.05.

**Table 2:** Exciton *g*-factors for monolayer and bilayer WSe_2_ from DFT.

Exciton	Valley (k_*c*_, k_*v*_)	Spin (*s*_*c*_, *s*_*v*_)	ML	BL, intralayer	BL, interlayer
*X*^0^	*KK*	↑↑	−4.0	−3.6	−13.2
*D*^0^	*KK*	↓↑	10.1	9.7	19.2
${K}_{L}^{\prime}$	$K^{\prime} K$	↑↑	13.6	13.2	3.6
${K}_{U}^{\prime}$	$K^{\prime} K$	↓↑	19.6	19.2	9.7
*Q*_*L*_	*QK*	↑↑	9.2	9.2	9.7
*Q*_*U*_	*QK*	↓↑	13.2	13.3	13.6
${Q}_{L}^{\prime}$	$Q^{\prime} K$	↑↑	10.2	9.7	9.2
${Q}_{U}^{\prime}$	$Q^{\prime} K$	↓↑	14.5	13.6	13.3
	$K \Gamma$	↑↑	5.8	5.8	3.7
	$K \, \Gamma$	↓↑	0.3	0.3	9.8
	$K^{\prime} \Gamma$	↑↑	3.7	3.7	5.8
	$K^{\prime} \Gamma$	↓↑	9.8	9.8	0.3
	$Q \, \Gamma$	↑↑	0.7	0.2	0.2
	$Q \, \Gamma$	↓↑	3.4	3.9	4.1
	$Q^{\prime} \Gamma$	↑↑	0.6	0.2	0.2
	$Q^{\prime} \Gamma$	↓↑	4.7	4.1	3.9

Exciton *g*-factors for selected spin-valley configurations of excitons in monolayer and bilayer WSe_2_. Note that without further assumptions the sign of the *g*-factor is meaningful only for zero-momentum spin-like excitons with valley-contrasting dipolar selection rules.

As evident from Fig. 4a, particular bands make decisive contributions to the *g*-factor. To discuss this behavior for the ML case in more detail, we consider the 24th and 26th bands that correspond to the highest valence band (*v*) and the second conduction band (*c* + 1), respectively, and give rise to largest mutual contributions in the *g*-factors. This is expected according to Eq. (5), where the orbital angular momentum is proportional to the product of the interband matrix elements, which in turn are largest for the fundamental A-exciton transition *X*^0^ between the 24th and 26th bands. Similar arguments apply for the mutual contributions of the 23rd and 25th bands to the *g*-factor of B-excitons. It is also instructive to note the different dependencies of the orbital momenta for the two lowest conduction bands (*L*_*c*_ and *L*_*c*+1_) and the top valence band (*L*_*v*_) on the number of bands included. In Fig. 4a, *L*_*c*_ and *L*_*c*+1_ exhibit jumps at 23rd and 24th bands, respectively, and then increase only marginally. In contrast, *L*_*v*_ in Fig. 4a increases nearly monotonously beginning from *m* ~ 30 on. A closer inspection shows that for *m* > 26, the sign of the square bracket in Eq. (5) alternates with increasing *m*, and the terms of comparable absolute values therefore cancel each other for both *L*_*c*_ and *L*_*c*+1_. For *L*_*v*_, on the other hand, the absolute values of the positive terms systematically exceed the negative terms and thus *L*_*v*_ continues to grow with increasing band number. Further analysis will be required to understand this behavior in more detail.

**Fig. 5:**
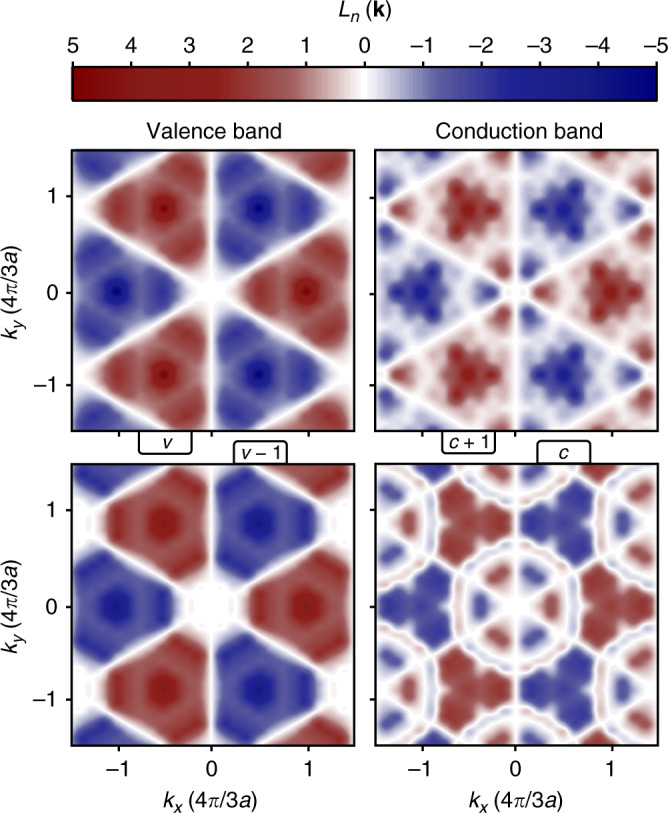
Orbital angular momentum of the highest valence bands and lowest conduction bands in monolayer WSe_2_ from DFT. Orbital angular momentum in the first Brillouin zone for the highest valence bands *v* and *v* − 1 (left panels) and lowest conduction bands *c* and *c* + 1 (right panels) from DFT calculations including spin–orbit effects.

The DFT results for *L*_*n*_(**k**) within the first Brillouin zone are shown in Fig. 5. Since spin–orbit effects were included at the DFT level, it is instructive to show both spin–orbit split highest valence bands (*v* and *v* − 1) and lowest conduction bands (*c* and *c* + 1). With the matrix elements of the orbital angular momenta of the valence and conduction bands in Fig. 5, it is straight forward to calculate the *g*-factors of the lowest-energy ML excitons in various configurations. In Table 2, we list the *g*-factors obtained from our DFT results for excitons in different configurations of valleys (**k**_*c*_, **k**_*v*_) and spins (**s**_*c*_, **s**_*v*_, with ↑ or ↓ projection along *z*).

In the top block of Table 2, we list excitons with the hole at *K* and the electron at *K* or $K^{\prime}$ in spin-like and spin-unlike configurations with short exciton notation for zero-momentum bright and dark neutral excitons *X*^0^ and *D*^0^ and their finite-momentum counterparts ${K}_{L}^{\prime}$ and ${K}_{U}^{\prime}$. The block below shows the results for the spin-like and spin-unlike *Q*-excitons with the electron in *Q* and the hole in *K*, followed by two blocks without short exciton notation for momentum-indirect excitons composed from electrons in *K* or *Q* and holes in Γ. Note that the sign of the *g*-factor can be determined without further assumptions only for *X*^0^ with established valley-contrasting dipolar selection rules. For Zeeman-split momentum-indirect excitons, on the other hand, the sign will depend on the symmetry of the actual phonons involved in phonon-assisted PL^34^. This is analogous to the case of spin-dark excitons *D*^0^ with linearly polarized in-plane zero-phonon emission^30^ contrasted by circularly polarized PL sidebands of the same reservoir mediated by chiral phonons^33,45^. In principle, not only the *g*-factor signs but also the absolute values of the respective PL peaks should be distinct due to different exciton–phonon coupling not accounted for in our model. However, we expect such higher-order corrections to be well below the resolution of our measurements.

## Discussion

First, we discuss the results of our calculations for excitons in ML WSe_2_. The *g*-factor of  −4.0 from our DFT model is in excellent agreement with the experimental value of  −4.1 for *X*^0^ (refs. ^12–17^). The good agreement in the *g*-factor of spin-dark excitons with *g* ≃ 9.4 in experiment^20^ and 10.1 in DFT provides further confidence in our model. According to our calculations, the states ${K}_{L}^{\prime}$ and ${K}_{U}^{\prime}$, which are the momentum-indirect counterparts of *X*^0^ and *D*^0^, exhibit different *g*-factors with large values of 13.6 and 19.6, respectively. The *g*-factors of *Q*-momentum excitons (9.2–14.5) are similar to those of *D*^0^ and ${K}_{L}^{\prime}$, whereas excitons with the hole at Γ are predicted to have rather small *g*-factors (<5.8) except for the spin-unlike configuration with $K^{\prime}$ electron (9.8). As expected, the *g*-factors of intralayer excitons in BL WSe_2_ are close to the values of the corresponding ML excitons^46^. In addition to intralayer excitons, the BL hosts interlayer counterparts (e.g., intralayer *Q*_*L*_ and interlayer ${Q}_{L}^{\prime}$, intralayer *Q*_*U*_ and interlayer ${Q}_{U}^{\prime}$, so on) that exhibit the same *g*-factors within our model, which neglects Coulomb corrections for intralayer and interlayer excitons.

By providing explicit *g*-factor values for momentum-indirect excitons, our DFT results complement the experimental observations in ML and BL WSe_2_. In the framework of neutral MLs, however, they do not resolve the ambiguity between the two competing explanations of the peak ${M}_{1}^{0}$. The assignment of the peak as a phonon sideband of *Q*-momentum excitons^21^, on the one hand, is consistent with the *g*-factors of 9.2 and 14.5 for *Q*_*L*_ and ${Q}_{U}^{\prime}$ states in Table 2 (note that *Q*_*U*_ and ${Q}_{L}^{\prime}$ excitons, 250 meV above degenerate *Q*_*L*_ and ${Q}_{U}^{\prime}$ states^47^, are irrelevant in this context) and the structured peak ${M}_{1}^{0}$ in Fig. 2 with a *g*-factor of 11.5. On the other hand, the interpretation of the peak as direct PL emission by momentum-dark ${K}_{L}^{\prime}$ excitons^34^ is also consistent with the theoretical *g*-factor of 13.6 from DFT. Our DFT results also identify *K*Γ and *Q*Γ with small *g*-factors as potential candidates to explain the bright PL peak between ${M}_{1}^{-}$ and *D*^−^ in the negatively charged regime of high-quality samples with narrow spectra^33,34^.

For the neutral BL, our results help to rule out *Q*Γ excitons and suggest spin-unlike interlayer *QK* and intralayer $Q^{\prime} K$ exciton reservoirs rather than $K^{\prime} \Gamma$ as a joint origin of phonon sidebands ${B}_{1}^{0}$, ${B}_{2}^{0}$, and ${B}_{3}^{0}$ (ref. ^11^). Whereas a detailed assignment of the neutral BL peaks to the specific reservoirs and phonon sidebands is yet to be developed, the values of the exciton *g*-factors in the charged regime can be understood, as in the ML case, by regarding the additional electron in the charged complex simply as a spectator to the Zeeman effect of the neutral finite-momentum exciton reservoir.

In summary, our work provides exciton *g*-factors for neutral and charged ML and BL WSe_2_ from both experiment and DFT. For ML WSe_2_, the *g*-factors obtained from first-principles calculations are in excellent quantitative agreement with previous reports and complement these studies by providing theoretical *g*-factors for momentum-indirect excitons in different configurations of spins and valleys. For BL WSe_2_, our work adds insight into the origin of PL peaks on the basis of theoretical *g*-factor values. In the broad context of research on layered semiconductors and their applications, the theoretical aspects of our work provide guidelines for magneto-optical studies of single-layer TMDs, homobilayer or heterobilayer systems, and other realizations of TMD-based van der Waals heterostructures.

Note: During the submission of our manuscript, we became aware of three related works on the theory of exciton *g*-factors in TMD MLs and heterostructures from first principles^48–50^.

## Methods

### Experimental methods

The field-effect heterostructure consisted of an exfoliated WSe_2_ crystal (HQ Graphene) with extended ML and BL regions encapsulated in hBN (NIMS). To control the charge doping, the crystal was contacted by a gold electrode deposited on a 50-nm-thick thermal silicon oxide layer of a p-doped silicon substrate. With the electrode grounded, a gate voltage applied to the highly doped silicon was used to control the doping level in ML and BL WSe_2_. The sample was mounted in a cryogenic confocal microscope and cooled down in a closed-cycle magneto-cryostat (attocube systems, attoDRY1000) with a base temperature of 3.2 K. The PL was excited at 1.85 eV with a few μW power of a continuous-wave laser diode focused to the diffraction-limited confocal excitation and detection spot of a low-temperature apochromatic objective (attocube systems, LT-APO/VISIR/0.82), dispersed with a monochromator (Roper Scientific, Acton SP2500), and detected with a nitrogen-cooled CCD (Roper Scientific, Spec 10:100BR/LN). Magneto-luminescence experiments were performed in Faraday configuration with a bi-directional solenoid at magnetic fields of up to 9 T.

### DFT calculations

DFT calculations were performed within the generalized gradient approximation with the PBEsol exchange-correlation functional^51^ as implemented in the Vienna ab initio simulation package. The van der Waals interactions were considered with the DFT-D3 method with Becke–Johnson damping^52,53^; the spin–orbit interaction was included at all stages. Elementary cells with a vacuum thickness of 30 Å were used in order to minimize interactions between periodic images. The atomic positions were relaxed with a cut-off energy of 400 eV until the change in the total energy was <10^−6^ eV. The band structure of ML (BL) was calculated on the Γ-centered $\overrightarrow{k}$ grid of 12 × 12 divisions with 300 (600) bands.

## Data Availability

The data that support the findings of this study are available from the corresponding authors upon reasonable request.
